# Editorial: Insights in experimental pharmacology and drug discovery: 2022

**DOI:** 10.3389/fphar.2023.1250936

**Published:** 2023-08-22

**Authors:** Alexandra Latini, Carmen Peralta Uroz, Andrés Trostchansky

**Affiliations:** ^1^ Biochemistry Department, LABOX, Federal University of Santa Catarina (UFSC), Florianopolis, Brazil; ^2^ August Pi i Sunyer Biomedical Research Institute (IDIBAPS), Barcelona, Spain; ^3^ Facultad de Medicina, Unidad Académica de Bioquímica, Universidad de la República (UdelaR), Montevideo, Uruguay

**Keywords:** experimental pharmacology, drug discovery, cancer, pain-drug therapy, tetrahydrobiopterin—BH4, neuroinflammation

The aim set for this Research Topic was to continue providing a platform where researchers from around the world can describe new advances in the pharmacological field, and update the current state of scientific knowledge in areas related to pharmacological and therapeutic developments in human diseases. Following the Research Topic of 2022 (Trostchansky and Salomone, 2022), which focused on the use of various natural compounds with potential pharmacological effects, the authors contributing to this edition describe advancements in Therapeutic Approaches for Cancer Treatment, Chronic Pain Management, and Hormone-Dependent Diseases. In this context, the Research Topic comprises 4 articles, out of which two are original research articles, and the other two are literature reviews.

Advancements in developing effective anticancer therapies have been significant, but challenges remain in certain cancers like oral cancer. Identifying novel therapeutic alternatives is crucial. Anoctamin 1 (ANO1) is an intracellular calcium-activated chloride ion channel that plays various roles in cancer growth and metastasis. Park et al. investigated the inhibitory effect of schisandrathera D, a novel compound derived from Schisandra sphenanthera, on the voltage-gated calcium-activated chloride channel Anoctamin 1 (ANO1) in prostate and oral cancers. The authors reported that Schisandrathera D effectively suppressed ANO1 activation. Interestingly, it did not affect intracellular calcium concentration induced by ATP or cystic fibrosis transmembrane conductance regulator activity induced by forskolin. Additionally, schisandrathera D gradually reduced ANO1 protein content and significantly decreased cell viability in ANO1-expressing cells compared to ANO1-knockout cells. Notably, schisandrathera D displayed a superior binding capacity to ANO1 protein compared to the known ANO1 inhibitor, Ani9. Furthermore, schisandrathera D increased caspase-3 and cleaved poly [ADP-ribose] polymerase 1 levels, indicating its anticancer effect through apoptosis induction. These findings highlight schisandrathera D as a potential anticancer agent that reduces ANO1 protein levels and exerts apoptosis-mediated effects in prostate and oral cancers. In summary, the authors stated that their results provided the foundation for developing safe and effective anticancer drugs based on schisandrathera D.

Continuing with the discussion of the pharmacological combat to different types of cancer, Lanišnik Rižner and Romano highlighted the current status and limitations of hormonal drugs used in hormone-dependent diseases, such as hormone-dependent cancers and endometriosis. While current treatment approaches primarily target estrogen receptors or at pre-receptor levels to inhibit estrogen activity weak effects and low therapeutic efficacy have been observed, demanding further investigation. The review also discusses the potential advantages of combined treatments targeting multiple enzymes involved in local estrogen formation to enhance therapeutic outcomes. Local estrogen formation and activity play critical roles in hormone-dependent cancers and benign conditions like endometriosis. Current treatment options for these diseases primarily target estrogen receptors and the pre-receptor levels, focusing on inhibiting local estrogen synthesis. Aromatase inhibitors, which inhibit the conversion of androgens to estrogens, have had extensive use for more than 40 years to treat postmenopausal breast cancer as well as used in clinical studies for endometrial, ovarian cancers, and endometriosis. Both steroidal and non-steroidal aromatase inhibitors have shown some promise. Moreover, showing the importance of targeting estrogen formation and metabolism in cancer treatment, sulfatase inhibitors that target the enzyme responsible for the hydrolysis of inactive estrogen-sulfates have entered clinical trials for the treatment of breast, endometrial cancers, and endometriosis, primarily demonstrating effects in breast cancer. Recently, inhibitors of 17-beta-hydroxysteroid dehydrogenase 1, an enzyme involved in the production of the potent estrogen estradiol, have shown encouraging results in preclinical studies and are now being evaluated in clinical trials for endometriosis. Thus, the authors reviewed and discussed an overview of the current use of hormonal drugs for major hormone-dependent diseases. The authors also discussed the mechanisms underlying the sometimes-observed weak effects and limited therapeutic efficacy of these drugs, highlighting the potential benefits of combined treatments targeting multiple enzymes involved in local estrogen formation or drugs with distinct therapeutic mechanisms.

The development of novel analgesics for chronic pain has encountered significant difficulties, emphasizing the need for new, effective, and safe targets. Indeed, the development of effective and safe analgesics for chronic pain has proven challenging, often due to limited efficacy and dose-limiting side effects. Excessive tetrahydrobiopterin (BH4) levels have been implicated in chronic pain (Staats Pires et al., 2020) based on diverse approaches, including gene expression profiling experiments and human genome-wide association studies. This association has been validated by numerous clinical and preclinical studies. BH4 serves as a vital cofactor for enzymes involved in aromatic amino acid hydroxylation, nitric oxide synthesis, and alkylglycerol monooxygenase activity. Deficiency in BH4 leads to various symptoms in the peripheral and central nervous systems. Therefore, an ideal therapeutic approach would involve blocking excessive BH4 production while preventing BH4 depletion. Indeed, Cronin et al. discussed that inhibiting sepiapterin reductase (SPR; an enzyme involved in BH4 biosynthesis) specifically in peripheral tissues (excluding the spinal cord and brain) represents an effective and safe strategy for alleviating chronic pain. The review discusses the role of different cell types in peripheral tissues that contribute to BH4 overproduction and pain hypersensitivity, highlighting their blockade as sufficient for pain relief. Importantly, based on their hypothesis, the authors explore the potential safety profile of peripherally restricted SPR inhibition based on human genetic data, alternative routes of BH4 production in various tissues and species, and the challenges of translatability from rodent models. Finally, they discuss potential formulation and molecular strategies to achieve potent and peripherally restricted SPR inhibition, not only for chronic pain but also for other disorders where excessive BH4 has been implicated.

Finally, the current Research Topic included an original article that presented the role of cyclic nucleotides, specifically 3′,5′-cyclic adenosine monophosphate (cAMP) and 3′,5′-cyclic guanosine monophosphate (cGMP), in fundamental brain functions, including learning and memory. Yanai et al. demonstrated that phosphodiesterase 3 (PDE3) regulated the cellular content of cAMP and cGMP, which is in agreement with previous studies showing that long-term administration of cilostazol, a PDE3 inhibitor, improved memory performance in aging mice. The authors presented new findings that demonstrate the potential of cilostazol to reverse age-related memory impairments in aged male mice. Cilostazol treatment for 1 month in mice with age-related memory impairments reversed declines in memory performance, reduced neuroinflammation, and increased glucose uptake in the brain. The findings indicate that age-related memory impairment in elderly male mice, which is dependent on cyclic nucleotide signaling, can potentially be reversed by inhibiting PDE3. This reversal of memory impairments associated with aging may occur within the central nervous system through cilostazol-enhanced recall or the strengthening of weak memories that may otherwise be difficult to retrieve.
